# Patients as Feedback Providers: Exploring Medical Students’ Credibility Judgments

**DOI:** 10.5334/pme.842

**Published:** 2023-04-12

**Authors:** M. C. L. Eijkelboom, R. A. M. de Kleijn, W. J. M. van Diemen, C. D. N. Maljaars, M. F. van der Schaaf, J. Frenkel

**Affiliations:** 1 Faculty of Medicine, Utrecht University, the Netherlands; 2 Department of Pediatrics, University Medical Center Utrecht, the Netherlands; 3 Utrecht Center for Research and Development of Health Professions Education, University Medical Center Utrecht, the Netherlands; 4 Freudenthal Institute, Utrecht University, the Netherlands

## Abstract

**Introduction:**

Patient feedback is becoming ever more important in medical education. Whether students engage with feedback is partly determined by how credible they think the feedback provider is. Despite its importance for feedback engagement, little is known about how medical students judge the credibility of patients. The purpose of this study was therefore to explore how medical students make credibility judgments regarding patients as feedback providers.

**Methods:**

This qualitative study builds upon McCroskey’s conceptualization of credibility as a three-dimensional construct comprising: competence, trustworthiness, and goodwill. Since credibility judgments are shaped by the context, we studied students’ credibility judgments in both a clinical and non-clinical context. Medical students were interviewed after receiving feedback from patients. Interviews were analyzed through template and causal network analysis.

**Results:**

Students based their credibility judgments of patients on multiple interacting arguments comprising all three dimensions of credibility. In estimating a patient’s credibility, students reasoned about aspects of the patient’s competence, trustworthiness, and goodwill. In both contexts students perceived elements of an educational alliance between themselves and patients, which could increase credibility. Yet, in the clinical context students reasoned that therapeutic goals of the relationship with patients might impede educational goals of the feedback interaction, which lowered credibility.

**Discussion:**

Students’ credibility judgments of patients were a weighing of multiple sometimes conflicting factors, within the context of relationships and their associated goals. Future research should explore how goals and roles can be discussed between students and patients to set the stage for open feedback conversations.

## Introduction

It is becoming widely recognized that patients can contribute to students’ learning, and the number of initiatives that involve patients in medical education is growing [1,2]. One way to learn from patients is through their feedback [3]. As the users of healthcare, patients can provide unique perspectives on medical performance. Therefore, their feedback can be complementary to faculty or peer feedback. This is illustrated by studies in which patients rate students or residents differently on the same consultation compared to faculty [4]. In order for patient feedback to contribute to student learning, students need to engage with this feedback, meaning they should seek it, make sense of it, and act upon it [5]. Whether students engage with feedback is partly determined by their judgment of the feedback provider’s credibility [5,6,7,8,9,10,11]. In accordance with this, a review by Baines *et al*. reports that the impact of patient feedback on doctors’ performance was influenced by doctors’ judgments of patients’ credibility [12]. However, little is known about how these judgments regarding patients’ credibility as feedback providers are made and on the basis of which arguments.

In line with Molloy and Bearman, we view credibility as an attribute of an individual, which is defined by judgments of others [13]. In this regard, credibility is not an absolute quality someone possesses, rather it depends on the perceptions of others. It is established in relationships with others and varies between these relationships [7,14,15]. So, whereas a patient can be seen as highly credible by one student, another student can judge the same patient as not credible. Credibility judgments are shaped by the context in which the judgment takes place, by its learning culture and the relationships that are formed within these contexts [7,16,17]. So, in theory, a student could judge a patient as highly credible in a classroom setting, but this same student might judge the same patient as not so credible in a hospital setting. Thus, credibility is dynamic and interpersonal. When a patient provides feedback to a medical student, this patient’s credibility is defined by that student’s judgment of that patient, in that context.

To further conceptualize credibility we build on the work of McCroskey, which is commonly applied in research on teacher credibility [18,19]. McCroskey defined and operationalized credibility as a three-dimensional construct consisting of Competence, Trustworthiness, and Goodwill [18]. Competence refers to someone having knowledge of, and expertise in, the subject of interest [20]. Trustworthiness refers to someone being of good character and being honest. Goodwill refers to whether someone cares about the receiver and has good intentions [18]. Thus, using this conceptualization of credibility in patient-student feedback interactions, students’ judgments of patients’ credibility should comprise judgments of patients’ Competence, Trustworthiness and Goodwill.

Although studies in education, and more recently in medical education, have shown that learners’ credibility judgments of teachers or supervisors are comprised of multiple elements, empirical studies on feedback interactions between patients and (future) healthcare providers mainly report single elements of credibility judgments [7,19]. In general, students hold positive judgments about patients as (potential) feedback providers [3,21,22,23,24], whereas doctors report both positive and negative judgments of patients as feedback providers [25,26,27]. The ability to observe performance is described as an argument positively contributing to patients’ credibility [26,28,29]. Arguments for reduced credibility, as mentioned by doctors or students, are: lack of medical knowledge, concerns about the sincerity of patients, and concerns about feedback being influenced by a patient’s diagnosis [8,24,27,30,31,32,33].

Medical students learn with and from patients in various contexts, such as the classroom, the hospital, a patient’s home or the community. As previously mentioned, credibility judgments are shaped by the context [7,16,17]. If we want medical students to engage with patient feedback, and thereby learn from patients, we need a better understanding of the process by which students make credibility judgments regarding patients, on which arguments they base these judgments, and how this is shaped by the context. This might reveal opportunities to improve students’ engagement with patient feedback. Therefore, this study aims to answer the following research question: How do medical students judge patients’ credibility as feedback providers in a clinical and in a non-clinical context?

## Methods

### Study design

We undertook a retrospective qualitative interview study to understand students’ credibility judgments of patients in feedback interactions. Ethical approval of this study was provided by the Dutch Association for Medical Education (NVMO, NERB file number: 2020.5.8 and NERB file number 2021.8.8).

### Context and participants

We explored students’ credibility judgments regarding patients in a clinical and a non-clinical context. In both contexts students collected feedback from patients through (online) one-on-one dialogues.

The non-clinical context was a six-week elective course, in which pairs of sixth-year medical students developed audiovisual patient information, a knowledge clip. The knowledge clips were short (often animated) videos, where for instance a congenital heart disease is explained. Students developed these knowledge clips in collaboration with a patient and a Communication and Information Sciences (CIS) student [34]. The goal for medical students was learn how to identify a patient’s information need, create understandable information, and collaborate with a patient and a CIS student. During the course, the pair of medical students met three times with the patient. In meeting 1, the medical students and the patient got acquainted and determined the subject of the knowledge clip. In meetings 2 and 3, medical students received patient feedback on their knowledge clip and cooperation skills. The medical student pair also met three times with the CIS student, who provided advice on how to develop audiovisual information and gave feedback on the knowledge clip [34]. In August 2020, twelve medical students from an academic hospital in the center of the Netherlands enrolled in this elective course. The course was entirely online, due to the COVID pandemic. Eleven out of twelve medical students who signed up for the course enrollees provided informed consent to participate in this study.

The clinical context was a twelve-week clerkship combining Pediatrics and Gynecology, in which fourth-year medical students participated as a mandatory part of their medical curriculum. During the clerkship, students asked two patients for feedback. In preparation, students completed a self-directed feedback course on asking for relevant feedback, at the right time, through dialogue [35]. At the end of the clerkship, students participated in a facilitated reflection session, which focused on sense-making and action-planning [35]. Students who completed their clerkship between June – August 2022, were asked to participate in this study. Ten students out of 54 provided informed consent to participate.

To ensure anonymity, and for the sake of clarity, all students in this paper will be referred to as ‘she’, and all patients will be referred to as ‘he’. For the sake of brevity, medical students will be referred to as students.

### Data collection

For the non-clinical context, we developed a semi-structured interview guide based on credibility literature. We piloted the interview guide with two students, which resulted in minor adjustments. Then, CE and NM conducted the first two interviews together to ensure subsequent interviews were conducted similarly and comparably. The remaining nine interviews were divided between NM and CE.

We adjusted the interview guide to fit the clinical context and build on the results from the non-clinical context. A third researcher (WD) performed most of the interviews from the clinical context. First, CE and WD conducted two interviews together to train WD in performing the interviews comparably to the non-clinical context. Then, WD conducted the remaining interviews.

The interview guide included questions on patients’ credibility, students’ relationship with the patients and the feedback messages (See Appendix A). Students were interviewed individually after completion of the elective course or clerkship. The interviews, which were conducted via video-call or face-to-face, were audio recorded and transcribed verbatim. Interviews lasted approximately 60 minutes.

### Data analysis

To understand how students make credibility judgments of patients, we analyzed their reasoning about credibility. We performed template analysis, which is form of thematic analysis, to identify students’ arguments for increased or reduced credibility [36]. We chose this method because it allowed for building on previous feedback credibility literature, and left room for inductively developing new codes regarding patient credibility. To understand how students built their judgment and how this was impacted by contextual elements, we performed a causal network analysis. We chose this method because it maps the coherence between variables of a process, which can also be applied to cognitive processes such as making a credibility judgment [37]. The elements in students’ reasoning for determining a patient’s credibility are called ‘arguments’ in this study.

#### Template analysis

The coding template was developed in three steps, using both an inductive and deductive approach. First, a priori codes were defined constituting arguments described in literature on which credibility judgments can be based (Appendix B) [6,10,11,14,15,18,19,38]. Second, interviews from the non-clinical context were used to construct the initial coding template. CE and NM iteratively applied the coding template to the interviews, whilst adding new codes and refining existing codes. To further refine and structure the codes, they discussed the coding template with RK after coding interviews 1–3, 4–5, and after interviews 5–12. Codes were structured according to the three credibility dimensions: Competence, Trustworthiness, and Goodwill [18]. Several codes did not fit the credibility dimensions. These included elements of the context and two emergent themes: the feedback message and previous experiences with patient feedback. Third, CE and WD applied the initial coding template to the first six interviews from the clinical context and modified the template by adding new codes and redefining existing codes. The modifications were discussed with RK to define the final coding template. Lastly, CE applied the final coding template to the full dataset and findings were discussed with the entire research team until consensus was reached about the final interpretation.

#### Causal network analysis

**Figure 1:**
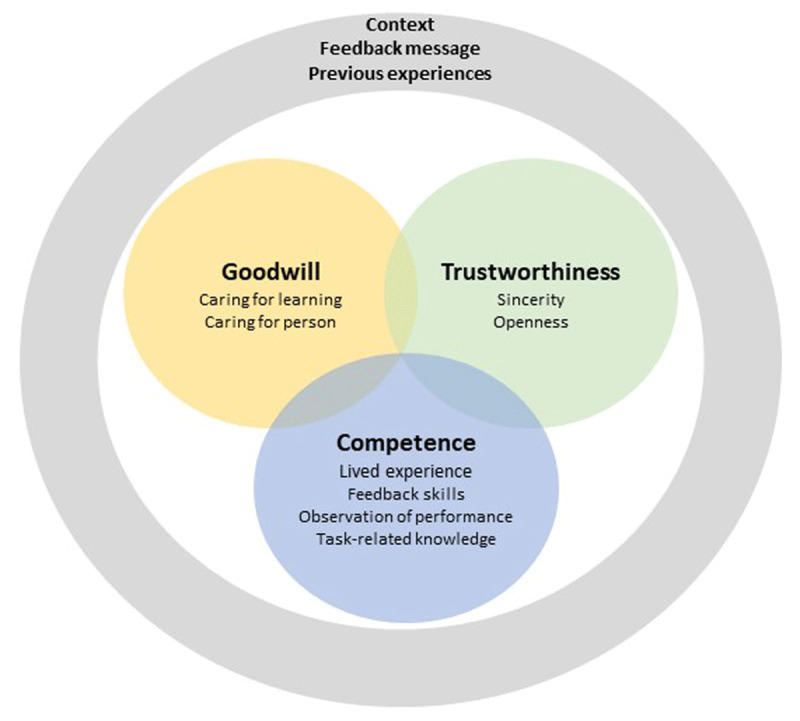
This figure visualizes students’ credibility judgments of patients as feedback providers. Students’ credibility judgments consisted of multiple arguments regarding a patient’s Goodwill, Trustworthiness and Competence. Arguments within and between the dimensions interacted with each other. Moreover, students’ credibility judgments were influenced by elements of the context, the feedback message and previous experiences, which is depicted by the gray circle. These elements could affect arguments regarding a patient’s Goodwill, Trustworthiness, and Competence.

To explore how judgments were built, and how arguments were related, we performed cross-case causal network analyses [37]. First, we constructed a list of all arguments on which students based their credibility judgments. This list was derived from the template analysis, each code of this codebook resembled an argument. Second, we constructed 21 causal networks, one for each individual student, in which we displayed these arguments and their relations. Relations were identified by selecting arguments that students mentioned in combination with each other (meaning text-fragments overlapped or followed-up on each other), by analyzing signal words (for instance: then, therefore, so, because), and by analyzing temporality (if one argument affects the other, this argument must happen first in students’ reasoning). Third, the 21 individual causal networks were combined in an overarching network, that comprised all interactions between individual arguments. To make this network comprehensible, we simplified the network by only illustrating the interactions between dimensions of credibility and the elements of the context, the feedback message, and previous experiences, which resulted in Figure 1.

### Reflexivity

The research team consisted of medical doctors (CE, JF), educational scientists (RK, MS), and students (WD, NM). The varied backgrounds and roles of our team provided multiple perspectives on our data. Our team has the viewpoint of patients being important partners in learning, and being legitimate feedback providers. To limit transferring our viewpoint to the study participants, and facilitate students in sharing their own viewpoints, we carefully formulated the interview questions. Some authors were involved in the development of the non-clinical course (CE, JF) and the feedback training in the clinical context (CE, JF, RK). During the study period they did not participate as teachers in these trainings.

Furthermore, to enhance reflexivity biweekly research meetings were held during data collection and analysis, in which interpretation of the data and underlying assumptions were discussed.

## Results

Twenty-one students were interviewed, out of which 11 students in the non-clinical and 10 in the clinical setting. Students based their credibility judgments of patients on multiple arguments, comprising all three dimensions of credibility [18]. In estimating a patient’s credibility, students reasoned about his Competence, Trustworthiness, and Goodwill. Students’ reasoning showed that their judgments were shaped by perceived elements of the context. Besides contextual elements, the perceived feedback message and student’s previous experiences with patient feedback also impacted their credibility judgments.

We will discuss how students built their credibility judgments by elaborating on the arguments that students provided for their credibility judgments, how these were shaped by the context, and how these were affected by the feedback message and their previous experiences.

### Arguments on which students based their credibility judgments

Students’ judgments about a patient’s credibility contained multiple arguments regarding a patient’s Competence, Trustworthiness, and Goodwill, see Table 1. Students judged patients’ Competence based on four arguments. First, they estimated their knowledge of the specific task. In the non-clinical context, this could be knowledge about audiovisual communication. In the clinical context, this could regard medical knowledge. For instance, students judged patients as more competent when they were in the medical profession. Second, students estimated patients’ knowledge of how the student performed, which patients for instance could have gained through direct observation. Third, students estimated whether patients were experienced and skilled in providing feedback. Lastly, students estimated patients’ lived experience. Most students regarded patients as more competent when they possessed more experiential knowledge. Conversely, some students reasoned that their ‘new’ patient was actually very credible because he had a fresh perspective.

**Table 1:** Arguments on which students based their credibility judgments of patients.


CATEGORY	ARGUMENT	DEFINITION: “THE STUDENT DESCRIBED THAT THE … SHAPED THE PATIENT’S CREDIBILITY”

**Competence**	Lived experience	patient having knowledge of living with disease and managing healthcare, derived through experience t

	Knowledge of performance	patient’s knowledge on how the student performed, gained by observation of, or collaboration with, the student

	Feedback skills	patient’s skills and experience in providing feedback

	Task-related knowledge	patient’s knowledge on the task itself, for instance biomedical or communicative knowledge (this does not include experience knowledge)

**Trustworthiness**	Sincerity	trust or distrust that the patient was honest in the feedback he provided

	Openness	trust or distrust that the patient dared to be open and not withhold feedback

**Goodwill**	Caring for person	patient’s caring for, and interest in, the student as a person

	Caring for learning	patient’s caring for the student’s learning process and being engaged in the educational project

“He was not familiar with the hospital and didn’t know about how things worked and so on. And I think that actually ensured that what he said came out really sincere. Also from this ignorance, he asked many questions like we don’t understand this, or we don’t understand that. That did mean that he really just says what he feels, … I found him more credible because of it.” – St4clinical (Lived experience)

Students judged patients’ Trustworthiness on two arguments. First, students estimated whether a patient was sincere and honest in his feedback. Second, students estimated a patient’s openness and whether he dared to mention points for improvement.

“In terms of [feedback on] our collaboration, it’s a bit lower, because his feedback was mostly positive and I am not sure if he was being 100% honest. I find that hard to estimate. And that’s why I don’t find him particularly credible, as a person, to give me feedback. I can’t quite sense whether he is being completely honest in his feedback.” –St6nonclinical (Sincerity)

Students judged patients’ Goodwill on two arguments. First, they estimated whether the patient had the intention to help the student learn, for instance by taking time to provide feedback and by being engaged in the educational project. Second, students estimated whether the patient cared for the student as a person, for instance by showing interest in the student’s personal life and by expressing gratefulness for the student’s presence. The latter could both increase and decrease credibility. Some students reasoned that a patient would avoid criticism to avoid hurting the student’s feelings.

“Well, he might think ‘she’s just a student, I don’t really want to hurt her. Yes, and she’s also just trying to do the best she can.’ He may not want to hurt me by saying anything negative.” –St4clinical (Caring for person)

Causal network analysis showed that students built their judgments by weighing multiple arguments. Arguments of different dimensions interacted with each other (See Figure 1). For instance, a student reasoned that the patient was sincere and open (Trustworthiness), since she assumed that the patient cared for her learning (Goodwill). Our results suggest that students’ judgments of a patient’s credibility were not ‘all or nothing’ judgments. Rather, students assigned a certain degree of credibility to a patient, taking into consideration arguments that contributed to credibility, and arguments that reduced credibility.

### How elements of the context affect credibility judgments

Students mentioned four contextual elements that affected their credibility judgments (See Table 2).

**Table 2:** Elements of the context that shaped students’ credibility judgments.


ARGUMENT	DEFINITION: “THE STUDENT DESCRIBES THAT THE … SHAPED THE PATIENT’S CREDIBILITY”

Therapeutic relationship	therapeutic relationship between the student and patient, with the patient being in need of care and the student being part of the team providing care

Emotional state	patient’s emotional state (calm, stressed, anxious, relieved)

Other providers	other feedback-providers during the educational course (peer, CIS-student, CIS-teacher)

Trusting bond	the presence or absence of a positive bond, characterized by mutual respect or trust


Three contextual elements were either mentioned by students in the clinical context, or by students in the non-clinical context.

First, in the clinical context, students mentioned that there was a therapeutic relationship between them and the patient, with the patient being in need of care and the student being part of the team that provides care. Students reasoned that this could have hindered the feedback process, since patients could perceive a power imbalance and feel dependent on them. Therefore, patients might not dare to be open and honest (Trustworthiness), which lowered their credibility.

“He said that later on too, that he wants to maintain the treatment relationship, because he does depend on us as well. And I think that’s why he also has in the back of his mind that he has to remain friendly with us, which is perhaps why he didn’t want to be too critical.”-St4clinical

Interestingly, one student mentioned that over time the patient became more aware of her role as a learner, which had put the therapeutic relationship more to the background, and increased the patient’s willingness to help the student learn (Goodwill).

“because of that, I think he knew more what an intern entailed. And I think that actually allowed him to give better feedback, because he was more like okay, so you’re really here to learn and you’re not my doctor, so you’re not going to set my treatment plan. So I want to help you with that [ i.e. learning].”-St7clinical

Second, in the clinical context, students mentioned that the patient’s emotional state impacted his credibility. Students reasoned that feedback messages were shaped by patients’ emotions. For example, if a patient was happy or relieved due to receiving good news, then his feedback would be more positive, and vice versa. Moreover, students reasoned that patients would not dare to provide critical feedback (Trustworthiness) if they felt vulnerable, for instance because they were in an unknown environment or were very sick. Besides, students mentioned that patients who appeared calm and at ease where more credible compared to patients who were stressed or nervous, because the latter might not be in the right state of mind to care about students’ learning (Goodwill) or observe the student and make judgments about her performance (Competence).

“Yes [this patient was] therefore less nervous, and therefore less emotional, with more space to think about how [I am] doing in this work environment. There is therefore also more space to look at the other person instead of focusing too much on yourself. In my opinion, he could therefore give better feedback and was more credible. And he was a bit more objective as well, I think.” – St6clinical

Third, students in the non-clinical context mentioned that feedback messages of other providers on their audiovisual patient information (the knowledge clip) affected the patient’s credibility. In the educational course, students also received feedback on their knowledge clip from healthcare professionals and peers. When the patient’s feedback added to, or was in accordance with, feedback from healthcare professionals or peers, the patient was seen as more credible.

“Actually, the tips given by [a psychologist] were exactly the same, or almost exactly the same as [the patient’s]. So that was always very similar. … which did reinforce [his credibility].” -St9nonclinical

One element of the context, ‘trusting bond’, was mentioned by students in both contexts. A bond, characterized by mutual respect and trust, could both increase or decrease credibility. Some students reasoned that their positive bond made patients care more about their learning (Goodwill) and made them dare to be more open (Trustworthiness). However, other students reasoned that their positive bond made patients less open about points for improvement (Trustworthiness), because they feared damaging the relationship.

“Maybe that somehow made him a little less credible, that I thought … we have a very good relationship, does he still dares to be strict, so to speak, does he still dare to come up with tips.” –St3nonclinical

### How the feedback message and previous experiences affect credibility judgments

Besides elements of the context, students reasoning also showed that the feedback message, and previous experiences with patient feedback, affected students’ credibility judgments. With regard to the feedback message, when students perceived the feedback message as specific, extensive, and containing both positive feedback and points for improvement, they reasoned that the patient cared for their learning (Goodwill), was open and honest (Trustworthiness), and that he was skilled in providing feedback (Competence). This also worked vice versa:

“his feedback was mostly positive and I am not sure if he was being 100% honest. I find that hard to estimate. And that’s why I don’t find him particularly credible, as a person, to give me feedback. I can’t quite sense whether he is being completely honest in his feedback.”–St6nonclinical (Sincerity)

With regard to personal characteristics, some students in the non-clinical context had positive past experiences with patient feedback. This made them more aware of the value of patient feedback, and therefore judge patients as more credible.

“I do think I have learnt through that earlier experience that there is a lot of value in the patient’s feedback, because the patient is ultimately the person you either do want to convey a message to or you want to reassure. That’s the person you want to achieve your objective with, so that’s also the person who can actually give you the best feedback on whether you achieved that goal and why or why not.” –St1nonclinical

Analysis of students’ reasoning about credibility clarified that their credibility judgments regarding patients is a weighing of multiple interacting arguments. These arguments are shaped by the context, the feedback message and previous experiences (See Figure 1). Appendix C displays the causal network of a student, which shows how she built her credibility judgment of a patient.

## Discussion

In this interview study, we explored medical students’ credibility judgments of patients as feedback providers in a clinical and non-clinical context. Students based their credibility judgments of patients on multiple interacting arguments, which were affected by elements of the context, the feedback message and personal characteristics. We will discuss how our results contribute to the literature and we discuss their educational implications

Our findings indicate that students’ credibility judgments of patients align with McCroskey’s three-dimensional credibility model [18]. We found that in determining credibility, students estimated various aspects of a patient’s Competence, Trustworthiness, and Goodwill towards the student. Interestingly, regarding the argument ‘lived experience’, depending on *what* feedback students were looking for, presence or absence of lived experience could both contribute to credibility. Many students reasoned that a prolonged medical history contributed to credibility, because experiential knowledge allowed patients to judge a student’s performance in comparison to other hospital visits. However, other students reasoned that new patients were actually more credible, because they could provide a fresh perspective on care. Similar to student-resident interactions, our results therefore suggest that decisions about patient credibility were content specific, meaning dependent on the feedback message that students were seeking [7].

For educational practice, McCroskey’s three-dimensional credibility model can be used to discuss credibility judgments with students, and to help them organize and understand their arguments. Insight into their reasoning about credibility might help students to become more feedback literate in terms of which kinds of patients can provide which kinds of feedback. We encourage future research to explore whether discussing credibility judgments with students will enable them to seek and use feedback information from patients more deliberately.

Our results illustrate how context shapes credibility judgments. Exploring both a clinical and non-clinical context, with different roles for students and patients, highlighted how aspects of their relationship affected credibility. Interestingly, our results seem to resonate with the Educational Alliance Framework [39], which is used to describe the supervisor and trainee relationship and to understand how the feedback process is affected by their relationship [7,40]. Our results suggest that this Framework also applies to student-patient interactions: in both contexts students described elements of an educational alliance between them and the patient, which affected their credibility judgment [39]. As seen in supervisor-resident interactions, credibility increased when the feedback provider (i.e. the patient) showed interest in the student as a person and demonstrated that they wanted to contribute to the student’s learning process [7]. However, in contrast to supervisor-resident interactions, a bond of trust could both increase and decrease credibility [7]. Whereas some students reasoned that patients would feel free to speak their mind, other students reasoned that patients might withhold critical feedback in order to avoid hurting the student’s feelings, and preserve the positive relationship. Possibly, these students feared so called friendship-marking, a phenomenon seen in peer feedback, in which students find it uncomfortable to grade friends or peers too harshly, which leads to over-marking [41].

The clinical context differed from the non-clinical context, since as well as being learners, students were also healthcare providers and patients were in the direct need of care. Thus, in addition to an educational alliance, students also acknowledged a therapeutic alliance [42]. Consequently, the primary goal of the student-patient relationship shifted from the student’s learning process to the patient’s care, which created a perceived shift in power. This perceived shift of goals and power often led to reduced patient credibility: although students believed patients were of goodwill since they wanted to help them learn, students questioned patients’ trustworthiness since they reasoned that patients might not dare to be open about points for improvement, out of fear of negatively impacting their future healthcare [31].

Our results illustrate that credibility judgments are not straightforward, but are the result of a complex weighing of multiple factors, within the context of relationships and their associated goals. In the clinical context, students mentioned a conflict between goals of the therapeutical alliance (caring) and goals of the educational alliance (learning). As shown by a student, role clarifications and goal conversations between students and patients can set the stage for open feedback conversations [40]. Future research should explore how goals and roles can be discussed in student-patient relationships, while respecting different patient perspectives on their role in feedback conversations and how this affects credibility judgments [43].

Lastly, we found that perceptions of feedback messages impacted credibility judgments. Students judged patients as less credible when they considered the feedback message uninformative, nonspecific, or containing mainly positive feedback, and vice versa. Associations between the feedback message and the feedback provider’s credibility are usually described as the credibility of the provider influencing the credibility of the message [7,44,45]. Our results, however, suggest that it is a loop. Consequently, we should not only view provider credibility as a prerequisite for engagement with feedback messages, but also as a consequence of engagement with feedback messages.

In practice, to enhance student engagement with patient feedback, we should strive to increase the quality of patients’ feedback messages by training students to collect informative feedback from patients. Since receiving non-specific and mainly positive feedback from patients is often reported [23,46,47,48], we suggest focusing this training on asking for informative feedback, by formulating specific questions and engaging in a dialogue [35,49]. Moreover, voluntary feedback training for patients, and feedback tools like questionnaires, could support the process [46,50,51]. Future research could explore how these training activities affect credibility judgments.

### Limitations and suggestions for future research

In this study we focused on students’ perceptions of their credibility judgments, which inevitably brings limitations. The study data is based on recall, which leaves the possibility that arguments for credibility remained hidden. Moreover, since we studied perceptions, external and subconscious aspects that can influence credibility judgments, like institutional culture, might also have remained hidden [52,53]. Experimental designs that modify and control these aspects could reveal their impact on credibility judgments.

Moreover, our study population might limit the transferability of our data to experienced healthcare professionals. For most students in the clinical context, it was their first time asking patients for feedback. Our results show that credibility judgments are partly shaped by past experiences of the feedback receiver. Therefore, experienced healthcare professionals might judge a patient’s credibility differently. However, our purpose was to conduct an in-depth exploration of students’ credibility judgments regarding patients. Our results therefore serve as a starting point on which future studies can build, to further unravel the complex process by which credibility judgments regarding patients are made by (future) healthcare professionals.

In conclusion, medical students judge aspects of patients’ Competence, Trustworthiness and Goodwill to determine whether they are credible feedback providers [18]. Students’ judgments are a weighing of multiple and sometimes conflicting factors, within the context of relationships and their associated goals. In the clinical context, perceived tensions between therapeutic goals and educational goals of the student-patient relationship can diminish credibility. Future research should explore how goals and roles can be discussed between students and patients to set the stage for open feedback conversations.

## Additional File

The additional file for this article can be found as follows:

10.5334/pme.842.s1 Appendices. Appendix A to C.
