# Supplementary material for: Patients as Feedback Providers: Exploring Medical Students’ Credibility Judgments
Source: Perspect Med Educ. 2023 Apr 12;12(1):129–40. doi: 10.5334/pme.842 (PMC10103723; doi:10.5334/pme.842)
Supplement: Appendices. — Appendix A to C. [file pme-12-1-842-s1.pdf]

Appendix A – Interview guides

For the interview guide in the clinical context we used the source credibility scale (question 4). This questionnaire measures students' perception of trustworthiness, competence and goodwill. The questionnaire was used as a tool to stimulate discussion, not to quantitatively measure students' credibility judgments, since this tool has not been validated in this context. (McCroskey, J. & Teven, J. Goodwill: A Reexamination of the Construct and its Measurement. Commun. Monogr. 6, 90–103 (1999).)

<u>Interview questions non-clinical context</u>

1. How would you describe the course to a friend?

2. During the course you cooperated with a patient. How would you describe this patient?
   - How would you describe your relationship with this patient?

3. At several moments during the course you received feedback from the patient. Could you indicate, in this timeline, how credible you judged this patient as feedback provider throughout the course. [student fills in timeline]
   - Why did you assign this level of credibility at the start of the course?
   - Why didn't you assign more credibility to the patient at that point in time?
   - Why didn't you assign less credibility to the patient at that point in time?
   - Why did the level of credibility change at … moment? What happened during that moment?
   - You mentioned ….[summarize the arguments] where there any other factors that shaped your credibility judgment?

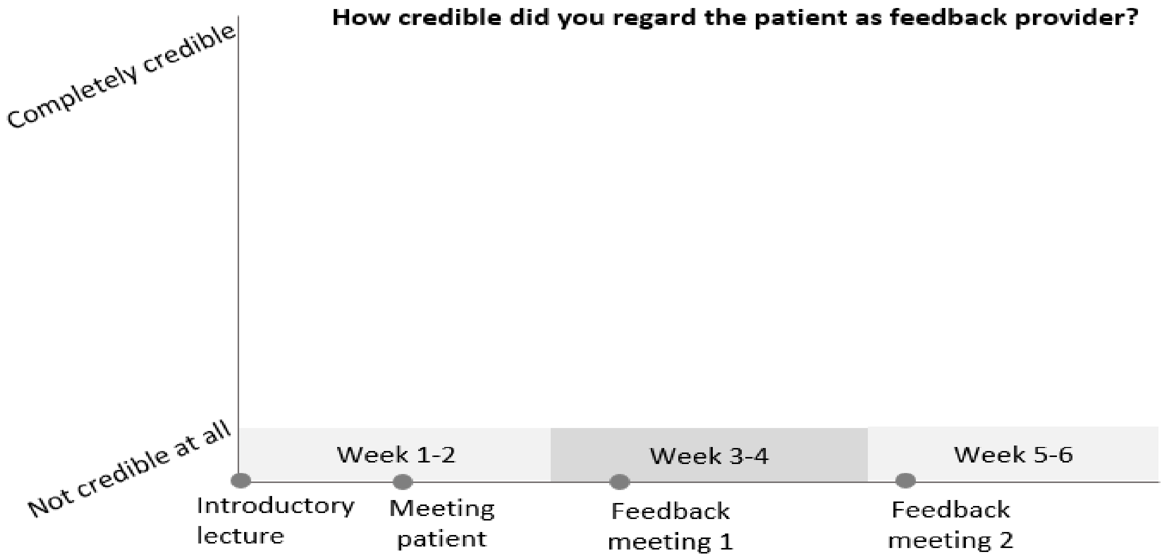

during

4. What feedback did you receive during meeting 1 [ask same question for meeting 2]
   - Did you consider this feedback useable? Why?
   - Was the patient the right person to provide you with this feedback? Why?

5. You just explained your relationship with the patient as …. Did this relationship change over time of the course?
   - Did your relationship influence the level of credibility you assigned to the patient over time of the course? How? (point at timeline)

6. Did you feel the patient had your best interest at heart?
    - Why yes? Why not?
    - Did this influence the level of credibility you assigned to the patient? How?

7. Did you have previous experience with asking patients for feedback?
    - If yes, please explain.
    - If yes, did this experience influence the way you judge patients as feedback providers? How?

8. Would you ask feedback from patients in the future?
    - Why?
    - If yes, on what aspects of your performance would you ask feedback from patients?

<u>Interview questions clinical context</u>

1. How many patients did you seek feedback from your clerkship?
    - How would you describe these patients?
    - How often did you see these patients?

2. What kind of feedback did you receive from the first patient? [repeat question for 2nd patient]

3. I am going to ask you some questions about the credibility of these patients as feedback providers. For each patient, please indicate on a scale of 1 to 10 how credible you find him or her. 1 is not credible at all and 10 is entirely credible.
    - Why did you assign this level of credibility to the patient?
    - Why didn't you assign more credibility to the patient?
    - Why didn't you assign less credibility to the patient?

4. You just indicated the level of credibility of the patients on a scale. Now you will be given a questionnaire on which you indicate which factors you included in your assessment of the patient's credibility.
    - You ticked ... factors (aspects about competence). Can you explain how these factors affected the credibility of the patients?
    - You ticked ... factors (aspects about goodwill). Can you explain how these factors affected the credibility of the patients?
    - You ticked ... factors (aspects about trustworthiness). Can you explain how these factors affected the credibility of the patients?

5. I'll now ask you a few short questions:
    - Did age/sex/occupation/social status play a role in determining credibility? How?
    - Did the patient's experiential knowledge play a role in determining credibility? How?
    - Did the patient appear to be engaged in your learning process? In what way? Did this affect the patient's credibility as a feedback giver?

6. How would you describe your relationship with the patient?
    - Did your relationship influence the level of credibility you assigned to the patient? How?

7. You indicated that you received .... as feedback. Did you find this feedback useful?
    - Did this affect how credible you found the patient? Can you explain that?

- Did you think the patient was the right person to give you this feedback? Why or why not?

8. What kind of environment (policlinic, ward, etc.) were you in at the time of asking for feedback?
   - Did this environment influence the feedback conversation you had?
   - Did this affect patient credibility? How?

9. You indicated that you had seen the patient ... times. Has your assessment of his or her credibility changed over time?
   - If so, how has their credibility changed? What is the reason for this?
   - Did your assessment of the credibility of each patient change after facilitated reflection session? How?

10. Did you have previous experience with asking patients for feedback?
    - If yes, please explain.
    - If yes, did this experience influence the way you judge patients as feedback providers? How?

Appendix B – Initial codebook with a priori codes

Code	Students described that … shaped the patient's credibility
Personality (patient)	character traits of the patient, for instance: friendliness, selfishness, extraversion, dynamism, humor
Age	the age of the patient
Gender	the gender of the patient
Power	the extent to which the patient can influence the student's assessment
Level of intelligence	the intelligence of the patient
Level of expertise/experience (patient)	the experience/expertise of the patient with the task itself
Level of informedness	whether the patient knows how the student performed on the task
Social status	the social status or professional background
Role model	the extent to which the student views the patient as a role model
Trust in intentions/motives	trust (or distrust) in the patient having good intentions or motives for giving feedback
Level of honesty	the feeling the patient is honest (or not) in his/her feedback message
Cultural background of patient	the cultural background of the patient
Attitude/behavior	the patient's attitude (nonverbal or verbal) towards the student. This includes, for example, paying attention, willingness to listen, showing respect, and valuing ideas
Personality (student)	student describes that own character traits play a role in determining credibility. (e.g. locus of control, self-esteem, position, motivation)
Level of experience (student)	the experience of the student with feedback
Prejudices of student	student describes that own prejudices regarding the patient play a role in determining credibility.
Familiarity with the patient (level of psychological closeness)	the familiarity with the patient (how well the students knows the patient)
Characteristics relationship	characteristics of the relationship (quality, duration, type, power relations, etc.)

Appendix C – Causal network of a student's credibility judgment

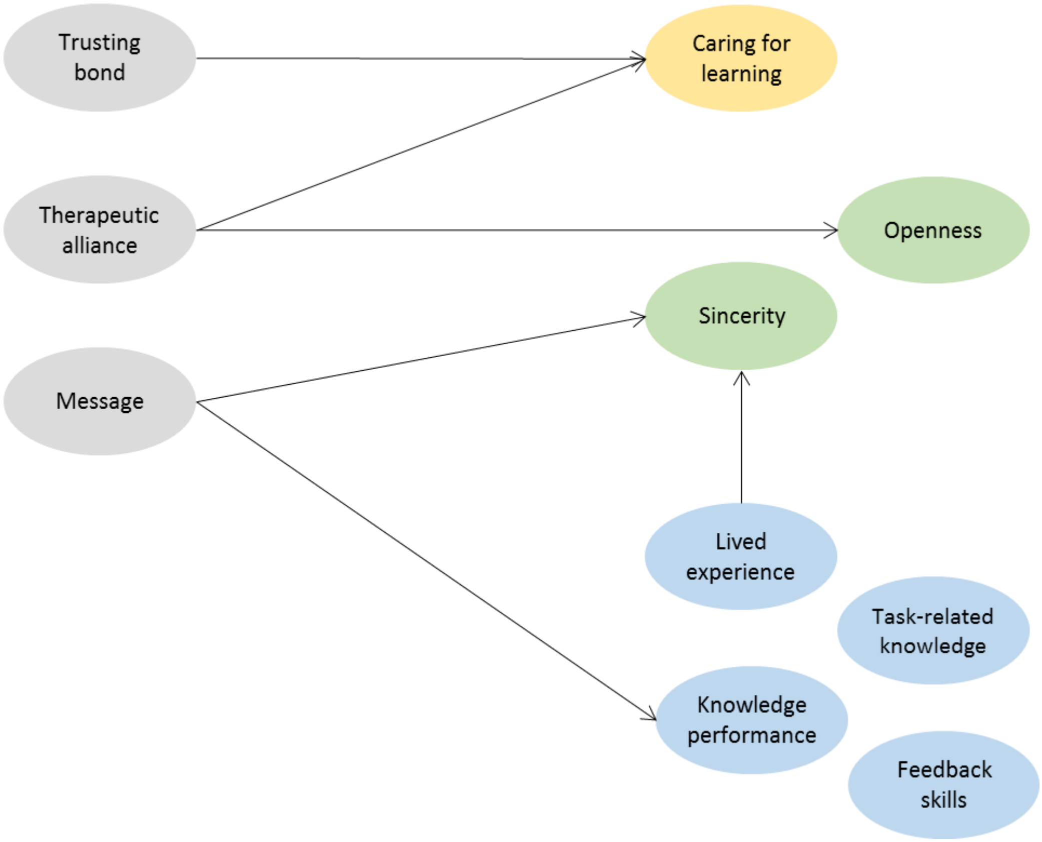

This student based her credibility judgment on 7 arguments, regarding the patient's Competence (blue), Trustworthiness (green) or Goodwill (yellow). Perceived elements of the context (the trusting bond and therapeutic alliance) and the feedback message affected the patient's perceived Competence, Trustworthiness and Goodwill.

Regarding Competence, the student felt the patient had a lot of lived experience, which made him capable of comparing the student's performance to other healthcare providers' performances. Moreover, the student considered the patient skilled in providing feedback because this was part of his profession. The student felt the patient had sufficient knowledge of her performance, since she had multiple interactions with this patient. This all increased credibility through competence. Yet, the student did not consider the patient fully competent, because the patient lacked task-specific biomedical knowledge. The student's judgment of the patient's competence was affected by the feedback message: the specific and elaborated feedback message made the student feel the patient really observed her, which increased his perceived competence:

> "So yes in the end, [the feedback message] does increase credibility. Because yes he really took a good look at what I found difficult in the conversation and ... really more comparing with the doctor and with me, like oh yes he explains things easier than you, so maybe you can do this differently." – St7

Regarding Trustworthiness, the student considered the patient sincere in the feedback he provided. This was positively affected by the feedback message and the patient's lived experience. The message contained both tips and tops, which made the student feel the patient was honest. Furthermore, the student reasoned that his many years of lived experience also

made him more frank. However, the student questioned whether the patient was completely open about points for improvement, because they had a therapeutic alliance.

> "Yes, I think patients will always be a bit cautious anyway, because they do want to maintain that treatment relationship" -St7

Regarding Goodwill, the student felt the patient cared for her learning since he took time to provide feedback and acknowledged her as a learner. The student developed a trusting and respectful bond with the patient, which further increased perceived caring for learning.

> "Because I did notice that there was just a good click, so I did feel that he was really trying to help me, so to speak."-St7

Interestingly, the student mentioned that throughout their relationship the patient became more aware of her role as a learner, which had put the therapeutic alliance more to the background and made the patient more willing to help the student learn.

> "because of that, I think he knew more what an intern entailed. And I think that actually allowed him to give better feedback, because he was more like okay, so you're really here to learn and you’re not my doctor, so you're not going to set my treatment plan. So I want to help you with that [e.i. learning].”-St7
